# The Expression Profiles and Clinical Significance of Mixed Lineage Kinases in Glioma

**DOI:** 10.1155/2024/5521016

**Published:** 2024-11-21

**Authors:** Jin Huang, Yuankun Liu, Gaosong Wang, Yuning Chen, Yifan Shen, Jiahao Zhang, Wei Ji, Junfei Shao

**Affiliations:** The Affiliated Wuxi People's Hospital of Nanjing Medical University, Wuxi People's Hospital, Wuxi Medical Center, Nanjing Medical University, Nanjing, China

**Keywords:** CGGA, glioma, mixed lineage kinase, prognosis, TCGA

## Abstract

Mixed lineage kinases (MLKs), comprising seven members: MLK1-4, dual leucine zipper kinase (DLK), leucine zipper kinase (LZK), and sterile alpha motif and leucine zipper containing kinase (ZAK), belong to the mitogen-activated protein kinase kinase kinase (MAP3K) family. These kinases are implicated in the progression of numerous cancers by activating mitogen-activated protein kinase (MAPK) cascades or functioning as ser/thr and tyr kinases. However, their specific roles in glioma remain elusive. In the present study, we utilized bioinformatics approaches to investigate the expression patterns of MLKs in low-grade gliomas (LGG) and glioblastoma multiforme (GBM). Additionally, we analyzed their clinical significance and delved into the potential mechanisms underlying MLK activity as well as their association with tumor-immune infiltrating cells (TIICs) in glioma. Furthermore, we conducted in vitro studies to elucidate the functional roles of MLK1-2 in glioma. Our findings revealed that the expressions of MLK1-2 were conspicuously downregulated in GBM and positively correlated with patients' overall survival. Conversely, ZAK exhibited an opposing trend. Notably, our newly devised risk score model exhibited superior performance in predicting patient prognoses. Moreover, we analyzed the potential mechanisms of MLK activity and its interplay with tumor immune infiltration. Last, we validated the antitumor effect of MLK1-2 at the in vitro level. In summary, our study sheds new insights into the roles of MLKs in glioma, particularly MLK1-2, and their potential as therapeutic targets.

## 1. Introduction

Glioma is one of the most prevalent and lethal malignancies among both children and adults [1]. Despite recent advancements in the treatment of glioma, the prognosis for patients remains poor [2]. The main factors limiting the prognosis of patients with glioma are the lack of precise prognostic biomarkers and effective therapeutic targets in clinical practice [3]. Therefore, it is of significant importance to develop novel prognostic indicators and valuable therapeutic targets for glioma.

Mixed lineage kinases (MLKs) are members of the mitogen-activated protein kinase kinase kinase (MAP3K) family. Based on sequence similarity within their structural and kinase domains, MLKs can be classified into three subclasses: the MLK core group (MLK1-4), the dual leucine zipper kinase (DLK) group (DLK and leucine zipper kinase (LZK)), and the sterile alpha motif and leucine zipper containing kinase (ZAK) group [4]. All MLKs possess a kinase domain and a leucine-zipper (LZ) domain. However, the MLK core group is characterized by an SRC homology 3 (SH3) domain on their N-terminus, along with a Cdc42/Rac interacting binding (CRIB) motif, followed by a large C-terminal region [5]. The DLK group possesses an additional LZ domain, while the ZAK group is distinguished by the presence of a sterile-alpha motif (SAM) immediately C-terminal to the LZ domain [6]. These structural differences between the three subgroups may underlie their distinct mechanisms of action.

In response to physical stress, drugs, inflammatory cytokines, or growth factors, MLKs can be activated by MAP4Ks, small proteins G, and oxidative stress [7, 8]. Once activated, MLKs proceed to activate multiple mitogen-activated protein kinase (MAPK) pathways. Additionally, studies have shown that MLKs can phosphorylate interacting proteins through protein–protein interactions (PPIs) [9]. MLKs are involved in a diverse array of physiological and pathophysiological processes, including cell proliferation, survival, differentiation, inflammation, metabolism, and apoptosis [10, 11].

Accumulating evidence also suggests that MLKs participate in tumor initiation and progression. For instance, mutations in the MLK1 gene have been identified in melanoma tumors [12] and H2009 nonsmall cell lung cancer cell lines [13]. The upregulation of MLK3 has been observed in various cancers, including breast, gastric, pancreatic, and ovarian cancer [14]. Overexpression of MLK3 promotes cell proliferation, cell cycle progression, migration, and invasion by activating multiple signaling pathways [7, 15]. MLK4 exhibits complex functions in cancer. In ovarian and colon cancer, MLK4 functions as a tumor suppressor gene [16, 17]. However, in colorectal cancer, hepatocellular carcinoma, and breast cancer, MLK4 promotes an invasive metastatic phenotype [18–20]. Furthermore, ZAK, another member of the MLK family, has been reported to possess both tumor suppressive and tumor-promoting activities [21].

MLKs have also been implicated in the pathogenesis of glioma. Specifically, knockdown of MLK3 was found to reduce the invasive aggressiveness of glioma cells by inhibiting estimated glomerular filtration rate (EGFR)-mediated jun N-terminal kinase (JNK) activation [22]. Moreover, ZAK expression was upregulated in glioma-exposed endothelial cells (GECs), where it enhanced blood–tumor barrier (BTB) permeability through the phosphorylation of nuclear factor kappa B (NF*κ*B)-p65 [23]. However, the potential application of other MLK family members in glioma, as well as the role of the entire MLK family, remains elusive. In our study, we comprehensively assessed the expression, clinical significance, correlation with tumor-immune infiltrating cells (TIICs), and possible mechanisms of MLKs in glioma using bioinformatics methods.

## 2. Materials and Methods

### 2.1. Gene Expression Analysis of MLKs in Public Databases

To analyze the expression of MLKs in various cancer types and their corresponding normal tissues, we utilized the Oncomine database, which comprises 715 datasets and 86,733 samples. We set a threshold of fold change = 2 and *p* value = 1E−4 to identify significant differences in MLK expression. Additionally, we downloaded the mRNA expression data and clinical relevance of MLKs in glioma from The Cancer Genome Atlas (TCGA) and the Chinese Glioma Genome Atlas (CGGA). Spearman's correlation coefficients were calculated to assess the correlations between the expression levels of different MLKs.

To visualize the expression level and spatial distribution of MLK proteins in normal brain tissues and glioma tissues, we employed the Human Protein Atlas (HPA) database [24]. This database contains immunohistochemistry and immunocytochemistry results for over 24,000 proteins, providing a comprehensive resource for protein expression analysis.

### 2.2. Genomic Alterations of MLKs in CBioPortal

CBioPortal [25] is a widely used open-access platform that enables researchers to explore, visualize, and analyze multidimensional cancer genomics data in an interactive manner. In this study, we utilized CBioPortal to investigate the genomic alterations, specifically mutations and copy number alterations of MLKs in glioma. By leveraging the extensive data available in CBioPortal, we aimed to gain insights into the potential role of MLK genomic alterations in glioma development and progression. The analysis provided us with a comprehensive overview of the frequency, type, and distribution of MLK mutations and copy number alterations in glioma patients, which may further our understanding of the molecular mechanisms underlying this disease.

### 2.3. RNA Extraction and Real-Time Quantitative PCR

Total RNA was extracted from the tissue samples using TRIzol reagent (Invitrogen, USA). Reverse transcription was performed using a PrimeScript RT reagent kit (TaKaRa, China). Quantitative PCR was then conducted using an SYBR green RT-PCR kit (TaKaRa, China) and specific primers (primer sequences are provided in the Table S1).

### 2.4. Clinical Significance Analysis of MLKs in Glioma

The mRNA expression levels of MLKs in glioma were analyzed for their correlation with clinical characteristics, including tumor grade, gender, age, and intradialytic hypotension (IDH) mutation status. This analysis was performed using data from TCGA and CGGA. Additionally, the least absolute shrinkage and selection operator (LASSO) regression analysis was employed to construct a new risk score model based on MLK expression data from TCGA. The prognostic value of this model was evaluated in both TCGA and CGGA datasets.

### 2.5. Correlation Between MLKs and Immune Infiltration

To investigate the potential relationship between MLK expression and immune infiltration in glioma, we utilized the Tumor Immune Estimation Resource (TIMER) 2.0 database [26]. This database allows for the exploration of correlations between gene expression and six types of TIICs: B cells, CD8 + T cells, CD4 + T cells, macrophages, neutrophils, and dendritic cells. We analyzed these correlations in both low-grade gliomas (LGG) and glioblastoma multiforme (GBM).

Furthermore, we employed TISIDB (http://cis.hku.hk/TISIDB), a web portal that integrates multiple heterogeneous data types related to tumor-immune system interactions [27]. Using TISDB, we analyzed the associations between MLK expression and lymphocyte populations in glioma.

### 2.6. PPI Network Via GeneMANIA Analysis

To gain insights into the potential interactions and functional associations of MLKs in glioma, we employed the GeneMANIA [28] tool to predict genes that may have interactions, coexpression, colocalization, and protein domain similarity with MLKs. The resulting PPI network provides a comprehensive overview of the biological functions and potential interactions of MLKs and their associated genes.

### 2.7. Functional and Pathway Enrichment Analysis Via Database for Annotation, Visualization, and Integrated Discovery (DAVID)

To further understand the biological significance of the genes identified in the PPI network, we performed functional and pathway enrichment analysis using the DAVID [29]. This analysis helps us identify the key biological processes (BPs), molecular functions (MFs), and pathways that are enriched in the gene set.

### 2.8. Western Blot Analysis

To validate the expression of MLKs and other proteins of interest at the protein level, we performed Western blot analysis. Total protein was extracted from glioma cells using radioimmunoprecipitation assay (RIPA) lysis buffer (CST, America). Protein concentration was measured using a bicinchoninic acid (BCA) protein assay kit (Thermo Fisher Scientific, Inc.). Equal amounts of protein samples were electrophoresed and transferred to poly(vinylidene fluoride; PVDF) membranes (Sigma, UK). The membranes were blocked with 5% skimmed milk for 1 h at room temperature and then incubated with primary antibodies against MLKs and other proteins of interest, as well as a secondary antibody against glyceraldehyde-3-phosphate dehydrogenase (GAPDH) as a loading control. The protein bands were detected using a FluorChem Q imaging system (ProteinSimple).

### 2.9. Colony Formation Assay

To assess the proliferative capacity of glioma cells, we performed a colony formation assay. Briefly, 1000 glioma cells were seeded into each well of a 6-well plate. After 12–14 days of culturing, the colonies were fixed with 4% paraformaldehyde and stained with 0.1% crystal violet. The plates were scanned, and the number of colonies was analyzed using ImageJ software. This assay provides a quantitative measure of the proliferative potential of glioma cells.

### 2.10. Migration and Invasion Assays

According to the manufacturer protocol, transwell plates (Corning, NY) were used to perform migration and invasion assays. For migration assay, 200 μL serum-free medium with 1 × 10^5^ indicated glioma cells were added to the upper chamber. For invasion assay, 40 μL matrigel medium (Corning, NY) was added to the upper chamber, followed by 160 μL serum-free medium with 1 × 10^5^ glioma cells. Then, 500 μL Dulbecco's modified eagle medium (DMEM) with 10% fetal bovine serum (FBS) was added to the lower compartments. After culturing for 24–48 h, the upper chambers were *t* immersed in 4% paraformaldehyde and stained with 0.1% crystal violet. The images were scanned by Invitrogen EVOS FL AUTO (Thermo Fisher).

### 2.11. Vector Construction and Transduction

Lentivirus containing Flag-*MAP3K10*-GV358 plasmids (MOI, 10) was synthesized by OBiO technology (Shanghai, China). Lentivirus containing Flag-*MAP3K9*-GV492 plasmids was purchased from Shanghai GeneChem (Shanghai, China). All transfections were conducted according to the manufacturer's instructions. Lipofectamine 3000 (Invitrogen) was used to conduct the transfections.

### 2.12. Statistical Analysis

Statistical analysis was performed by using IBM statistical package for the social sciences (SPSS) statistics (version 20.0). GraphPad Prism software (version 5.0) and R language (version 4.1.0) were used for figure generation. The measurement data were analyzed using a student's *t* test. Survival analysis was performed using log-rank tests. The clinical significance of MLKs was analyzed using the Cox regression model. A *p*  < 0.05 indicated a statistically significant difference.

## 3. Results

### 3.1. Expression Levels of MLKs in Glioma

**Figure 1:**
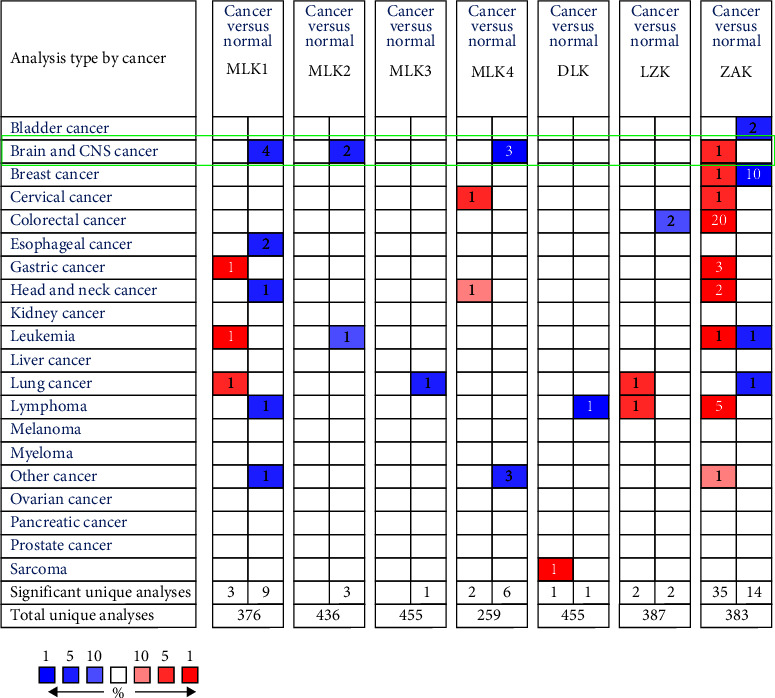
The expression of *MLKs* in pan-cancers (Oncomine). The search criteria were fold change = 2, *p* value = 1E−4. The cell number is the number of datasets that conform to the criteria. The color intensity (blue or red) represents the degree of downregulation or upregulation, respectively. The expression of *MLKs* in glioma was delineated with green highlights. MLK, mixed lineage kinases.

Utilizing the Oncomine database, we analyzed the mRNA levels of MLKs across pan-cancers (Figure 1 and Table 1). According to Sun et al., the expression of MLK1, MLK2, and MLK4 was significantly downregulated in glioma (comprising glioblastoma, anaplastic astrocytoma, and oligodendroglioma) compared to normal brain tissues. Conversely, Murat et al. reported an upregulation of ZAK expression in glioblastoma relative to normal brain tissues. Notably, the expression levels of MLK3, DLK, and LZK in Oncomine were not reported.

**Figure 2:**
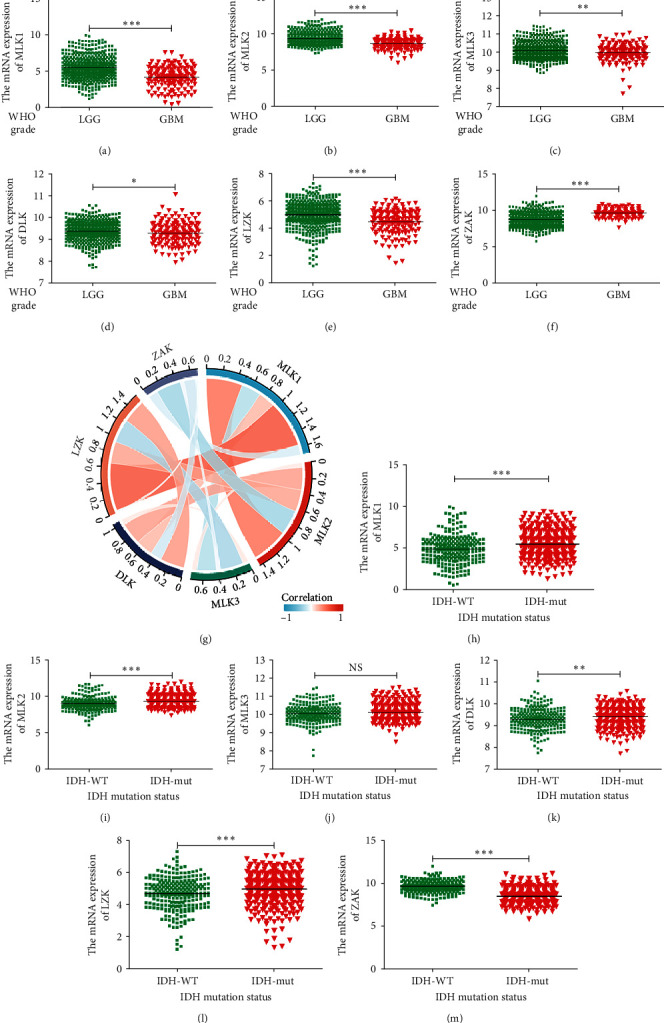
The mRNA expression levels of *MLKs* in glioma (TCGA). (A–F) The expression of *MLKs* in LGG and GBM (⁣^*∗*^*p* < 0.05, ⁣^*∗∗*^*p* < 0.01, and ⁣^*∗∗∗*^*p* < 0.001). (G) Coexpression chordal graph of *MLKs* in glioma. (H–M) The expression of *MLKs* in glioma with different IDH mutation states (⁣^*∗∗*^*p* < 0.01 and ⁣^*∗∗∗*^*p* < 0.001). GBM, glioblastoma multiforme; IDH, intradialytic hypotension; LGG, low-grade gliomas; MLK, mixed lineage kinases; TCGA, The Cancer Genome Atlas.

Analysis of the TCGA database revealed that the mRNA expression of MLK1-2, LZK, and DLK was significantly elevated in LGG compared to GBM, and these expressions positively correlated with IDH mutations (Figure 2A, B, D, E, H, I, L, M). Additionally, MLK3 expression was significantly higher in LGG than in GBM, but did not exhibit a correlation with IDH mutation type (Figure 2C, J). Interestingly, ZAK mRNA expression was higher in GBM and the IDH-wildtype group (Figure 2F, M). However, MLK4 expression was not reported in the TCGA database.

We further analyzed the Spearman's correlations between the mRNA expressions of MLK family members in TCGA and graphically depicted the results as a Chordal graph (Figure 2G, Table S2). Notably, we observed a positive correlation between the expression of MLK1 and MLK2 (cor = 0.479, *p*  < 0.001).

**Figure 3:**
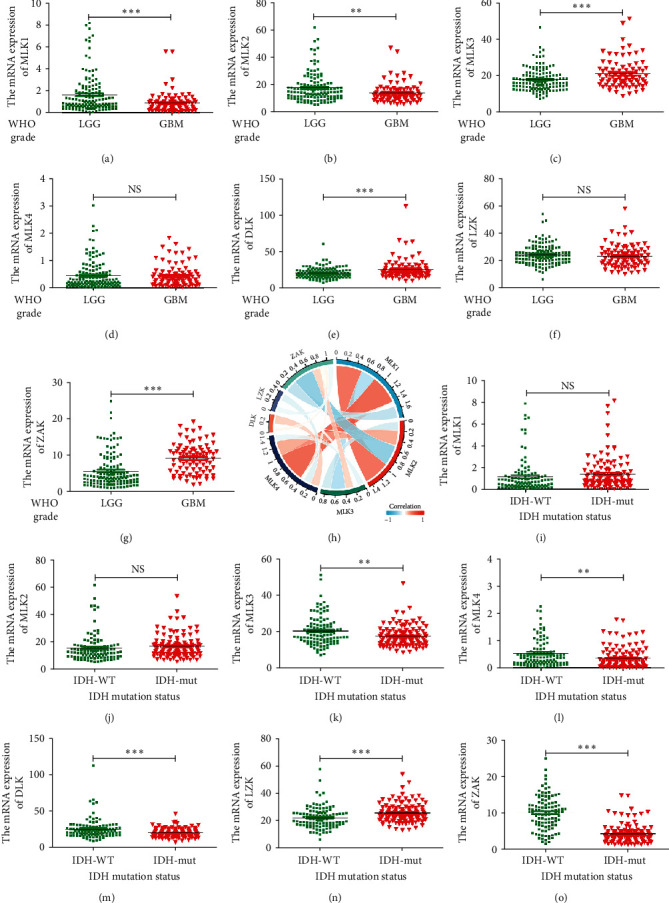
The mRNA expression levels of *MLKs* in glioma (CGGA). (A–G) The expression of *MLKs* in LGG and GBM (⁣^*∗∗*^*p* < 0.01, ⁣^*∗∗∗*^*p* < 0.001). (H) Coexpression chordal graph of *MLKs* in glioma. (I–O) The expression of *MLKs* in glioma with different IDH mutation states (⁣^*∗∗*^*p* < 0.01 and ⁣^*∗∗∗*^*p* < 0.001). CGGA, Chinese Glioma Genome Atlas; GBM, glioblastoma multiforme; IDH, intradialytic hypotension; LGG, low-grade gliomas; MLK, mixed lineage kinases.

Data from the CGGA database indicated that the expression of MLK1-2 was higher in LGG compared to GBM, yet this expression did not correlate with IDH mutation type (Figure 3A, B, I, J). Conversely, the mRNA expression levels of MLK3, DLK, and ZAK were higher in GBM than in LGG, and these expressions negatively correlated with IDH mutation type (Figure 3C, E, G, K, M, O). These findings provide insights into the differential expression patterns of MLK family members in glioma subtypes and their potential associations with molecular markers such as IDH mutations.

**Figure 4:**
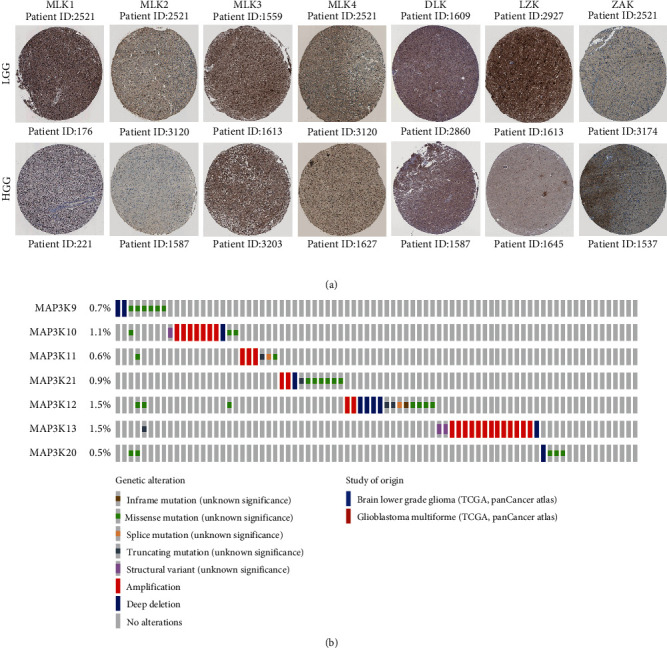
The protein level and genetic variations of MLKs in glioma. (A) The protein level of MLKs in LGG and HGG from the Human Protein Atlas. (B) Analyses of genetic variations of MLKs in glioma. HGG, high-grade gliomas; LGG, low-grade gliomas; MLK, mixed lineage kinases.

Then, we downloaded the protein level and genetic variation of MLKs in glioma from the Human Protein Atlas. The analysis results showed that the expression levels of MLK1, MLK2, MLK4 and DLK in LGG were higher than those in high-grade gliomas (HGG), while the expressions of MLK3 and ZAK were lower than those in HGG (Figure 4A). The genetic variation analysis diagram of MLKs shows the genetic mutations of MLKs in LGG and GBM (Figure 4B).

### 3.2. Clinical Relevance of MLKs in Glioma

To assess the clinical relevance of MLKs in glioma, we conducted an analysis examining the correlation between MLK expression levels and various clinical characteristics, including age, gender, World Health Organization (WHO) grades, and IDH mutation status (Tables S2 and S3). Our findings revealed that MLK1 and DLK did not exhibit significant correlations with age or gender, but they were notably associated with WHO grades and IDH-type in the TCGA dataset. However, in the CGGA dataset, only DLK displayed a similar pattern of correlation (Table S4).

**Figure 5:**
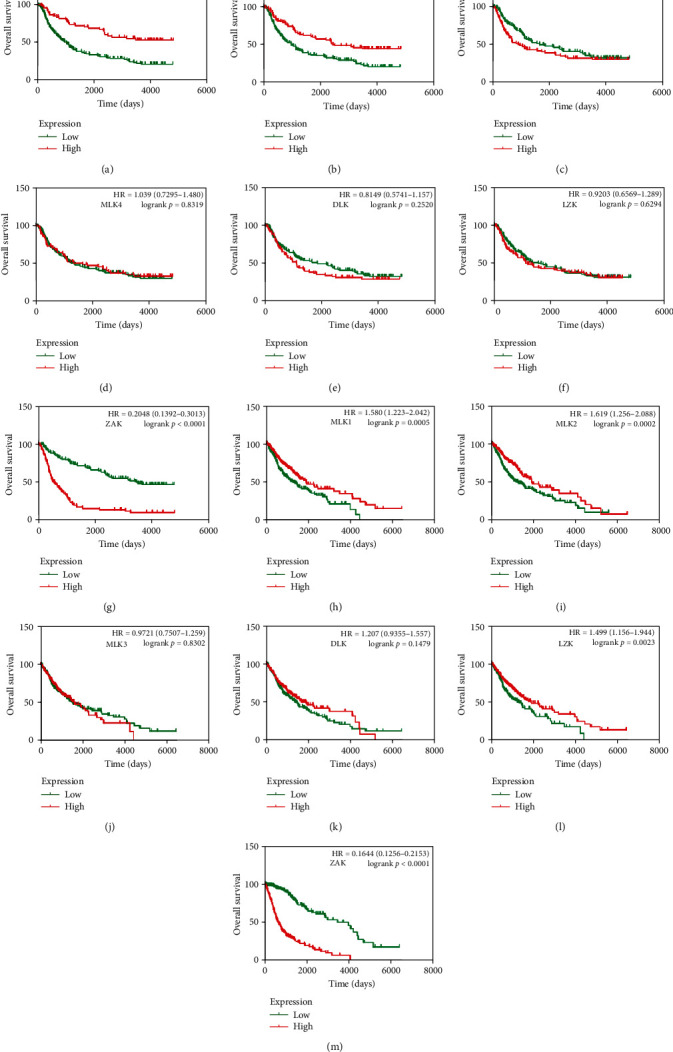
Prognostic value of MLKs mRNA expression in glioma patients (OS). (A–G) Prognostic significance of individual MLKs in glioma (CGGA). (H–M) Prognostic significance of individual MLKs in glioma (TCGA). The *p* values were calculated using the log-rank test. CGGA, Chinese Glioma Genome Atlas; MLKs, mixed lineage kinases; TCGA, The Cancer Genome Atlas.

Furthermore, to determine the prognostic significance of MLKs in glioma, we performed survival analyses in both TCGA and CGGA. As depicted in Figure 5, low expression levels of MLK1 and MLK2, as well as high expression of ZAK, were predictive of poorer clinical outcomes in both datasets (*p*  < 0.05). Conversely, the expression levels of MLK3, MLK4, and DLK did not exhibit significant correlations with patient prognosis. Notably, low expression of LZK was associated with a poorer prognosis in the TCGA dataset, though this finding was not significant in the CGGA dataset.

Additionally, we conducted subgroup analyses to further validate the prognostic relevance of MLKs in LGG and GBM (Tables 2 and 3). These analyses provided further insights into the potential clinical significance of MLK expression in specific glioma subtypes.

### 3.3. Construction of a Novel Prognostic Model for Glioma

**Figure 6:**
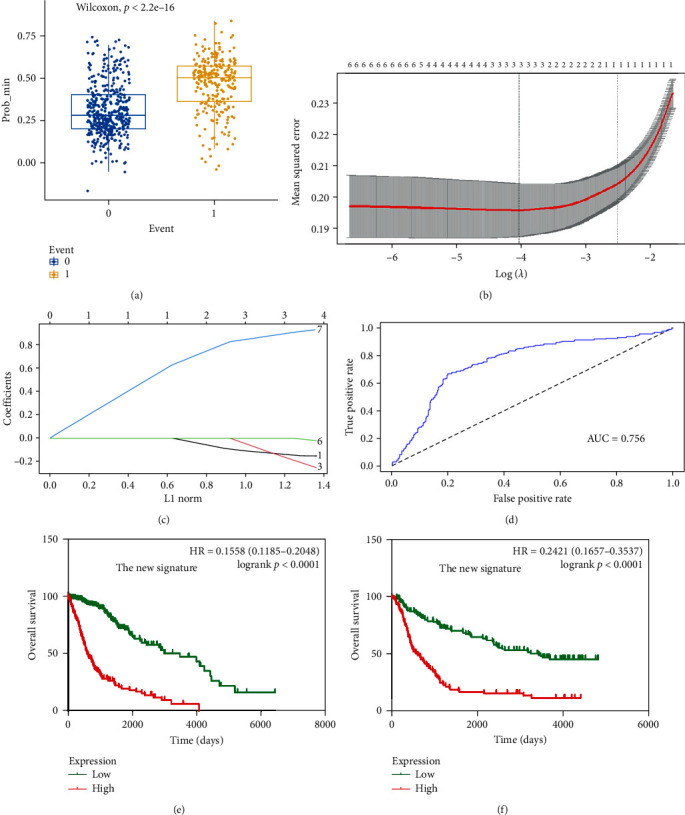
Construction of a new prognostic gene signature model. (A–D) Construction of a new prognostic signature. (E) The prognostic value of the new model in glioma demonstrated by TCGA. (F) The prognostic value of the new model in glioma by analyzing CGGA. CGGA, Chinese Glioma Genome Atlas; TCGA, The Cancer Genome Atlas.

Given the discrepancies in results obtained from the TCGA and CGGA datasets, we aimed to develop a new prognostic gene signature capable of more precisely predicting the prognosis of glioma patients. Utilizing data from TCGA, we performed LASSO regression analysis to establish a novel prognostic model. The risk score of this model is calculated as follows: risk score = expression of ZAK × 0.17814232–expression of MLK1 × 0.02817027–expression of MLK3 × 0.02165476. The discriminatory power of this model was assessed using the area under the receiver operating characteristic curve (AUC), which yielded a value of 0.756 (Figures 6A–D).

Subsequently, we examined the correlation between this novel signature and the prognosis of glioma patients in both TCGA and CGGA datasets. Our analysis revealed that patients belonging to the high-risk group exhibited significantly poorer prognoses compared to those in the low-risk group, both in TCGA and CGGA (Figure 6E, F). Furthermore, we analyzed the relationship between this new signature and various clinical characteristics (Tables S2 and S3), the results showed that this new signature was significantly associated with glioma grades and IDH type in both TCGA and CGGA.

Last, we conducted subgroup analyses to assess the performance of this new prognostic signature in specific subgroups. Our results indicate that this novel signature exhibits superior performance compared to previous models (Tables 2 and 3).

### 3.4. The Role of MLKs and TIICs in Glioma

**Figure 7:**
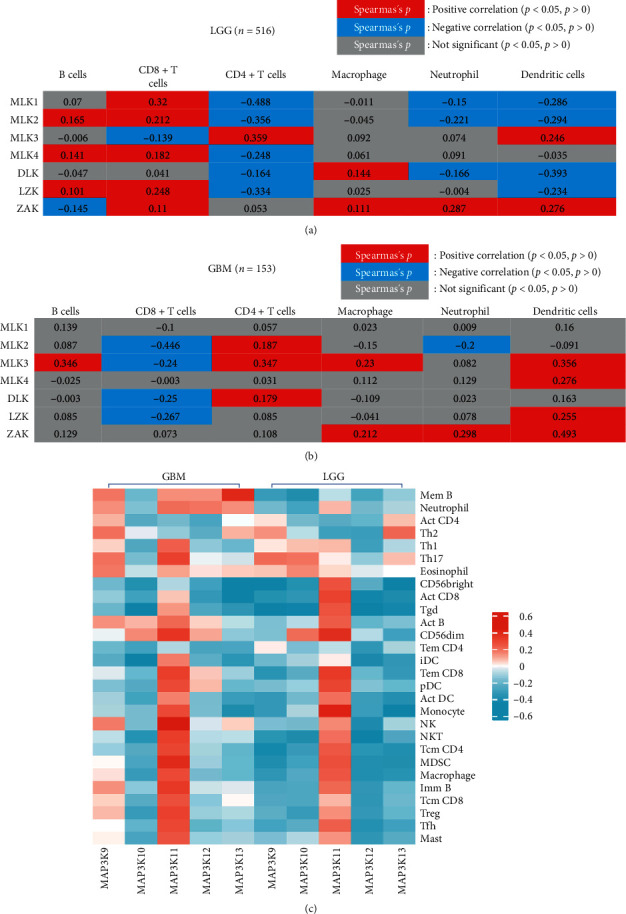
The association between MLKs and TIICs. (A) Correlation analysis between MLKs and six types of TIICs (B cells, CD8 + T cells, CD4 + T cells, macrophages, neutrophils, and dendritic cells) in LGG (*n* = 516); (B) Correlation between MLKs and TIICs in GBM (*n* = 153). The color of the cells represents statistical correlation (red means positive correlation, Spearman's *p*  < 0.05, blue means negative correlation, Spearman's *p*  < 0.05, grey means not significant correlation, Spearman's *p* ≥ 0.05). The cell number means coefficients for correlation between TIICs infiltration score and MLKs. (C) TISIDB database was used to analyze the association between MLKs expression and TILs. GBM, glioblastoma multiforme; LGG, low-grade gliomas; MLK, mixed lineage kinases; TIIC, tumor-immune infiltrating cells; TILs, tumor infiltrating lymphocytes.

Our analysis using TIMER 2.0 revealed interesting correlations between MLK expression and TIIC infiltration in LGG and GBM. For example, B cells were positively correlated with the expression of MLK2, MLK4, and LZK in LGG, but negatively correlated with ZAK. In GBM, B cells were positively correlated with MLK3 expression. CD8 + T cells showed positive correlations with MLK1, MLK2, MLK4, LZK, and ZAK in LGG, but negative correlations with MLK3. In GBM, CD8 + T cells were negatively correlated with MLK2, MLK3, DLK, and LZK (Figure 7A, B). These findings suggest that MLK expression may differentially affect the infiltration of specific TIIC subsets in LGG and GBM.

Furthermore, our analysis using the TISIDB database revealed significant correlations between MLK3 expression and the abundance of specific tumor-infiltrating lymphocytes (TILs) in GBM and LGG. Specifically, MLK3 was positively correlated with the abundance of natural killer (NK) cells, CD56 dim cells, and Th17 cells in GBM, as well as monocyte abundance in LGG. In contrast, MAP3K12 (another member of the MLK family) was negatively correlated with almost all TILs (Figure 7C).

Collectively, these results suggest that MLKs play important roles in shaping the tumor microenvironment of glioma by modulating the infiltration and function of TIICs. Further studies are needed to elucidate the specific mechanisms underlying these interactions and to explore potential therapeutic strategies targeting MLKs and TIICs in glioma.

### 3.5. Functional and Pathway Enrichment Analysis of MLKs in Glioma

**Figure 8:**
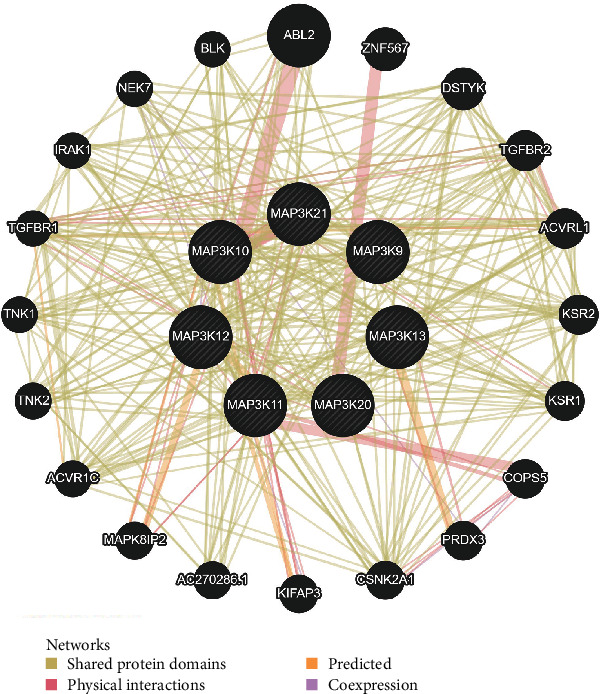
Protein–protein interaction network of MLKs and their coexpressed genes (GeneMANIA). The colors of the lines in the network represent the bioinformatics methods applied: shared protein domains, physical interactions, predicted and coexpression. MLKs, mixed lineage kinases.

Utilizing GeneMANIA, we predicted genes that are coexpressed with members of the MLK family, resulting in the identification of 20 genes (Figure 8). These genes, along with the seven members of the MLK family, were subsequently subjected to functional and pathway enrichment analysis using DAVID. As depicted in Table 4, the top five enriched MF terms were protein serine/threonine kinase activity, ATP binding, protein kinase activity, protein tyrosine kinase activity, and MAP kinase kinase kinase activity. Among the BP terms, the most significant enrichments were observed for protein phosphorylation, JNK cascade, protein autophosphorylation, peptidyl-threonine phosphorylation, and peptidyl-tyrosine autophosphorylation (Table 4). The top two enriched cellular component (CC) terms were intracellular and extrinsic components of the cytoplasmic side of the plasma membrane. Finally, the top five enriched Kyoto Encyclopedia of Genes and Genomes (KEGG) pathways were MAPK signaling pathway, adherens junction, TGF-β signaling pathway, Chagas disease (American trypanosomiasis), and Ras signaling pathway (Table 5).

These findings provide valuable insights into the potential roles of MLKs and their coexpressed genes in glioma progression, particularly in terms of their involvement in critical signaling pathways and cellular processes.

### 3.6. Verification of MLK1 and MLK2 Expression and Functional Analysis in Glioma

The consistency in the expression patterns and prognostic values of MLK1, MLK2, and ZAK (also known as MRK) between the TCGA and CGGA datasets prompted further investigation into the specific roles of MLK1 and MLK2 in glioma. Although previous studies reported antitumor effects of ZAK knockdown in GBM and medulloblastoma [30, 31], we sought to verify the expression and molecular function of MLK1 and MLK2 specifically in glioma.

**Figure 9:**
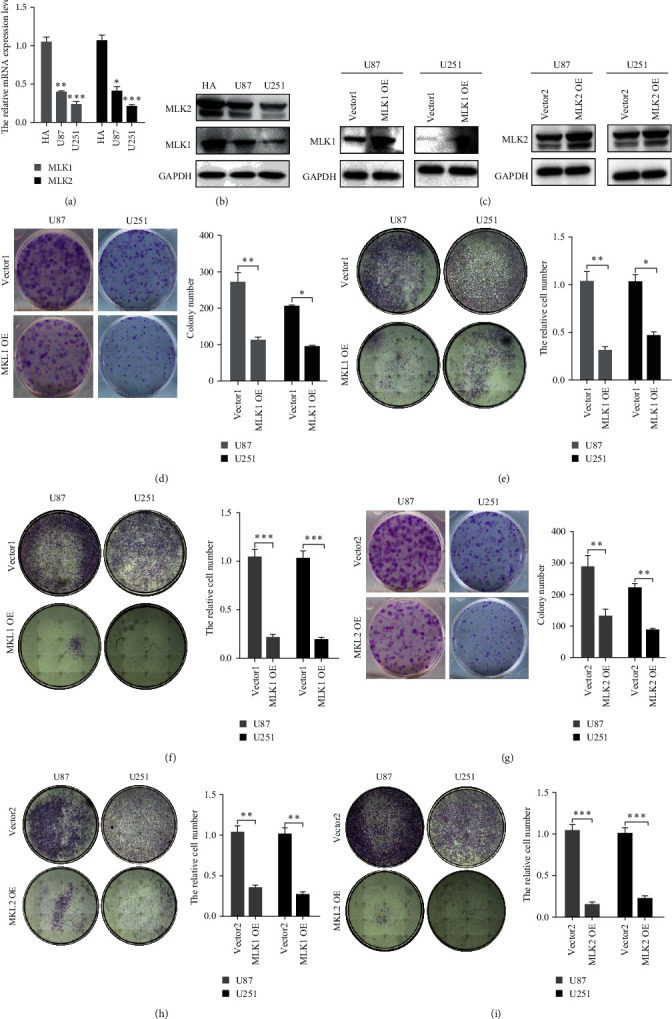
The expression and function of *MLK1-2* in glioma. (A**)** The protein level of *MLK1-2* in HA, U87 and U251 cells (⁣^*∗*^*p* < 0.05, ⁣^*∗∗*^*p* < 0.01, and ⁣^*∗∗∗*^*p* < 0.001). (B**)** The protein level of *MLK1-2* in HA, U87 and U251 cells. (C**)** Overexpression of *MLK1* and *MLK2* in U87 and U251 cells. (D–F**)** Overexpression of *MLK1* in U87 and U251 cells inhibited cell proliferation, migration, and invasion (⁣^*∗*^*p* < 0.05, ⁣^*∗∗*^*p* < 0.01 and ⁣^*∗∗∗*^*p* < 0.001). (G–I) Overexpression of *MLK2* in U87 and U251 cells inhibited cell proliferation, migration, and invasion (⁣^*∗*^*p* < 0.05, ⁣^*∗∗*^*p* < 0.01, and ⁣^*∗∗∗*^*p* < 0.001). HA, human astrocyte; MLKs, mixed lineage kinases.

Furthermore, the protein and mRNA levels of MLK1-2 were notably lower in glioma cells (U87 and U251) compared to normal human astrocytes (HA) (Figure 9A, B).

To further elucidate the functional role of MLK1 and MLK2 in glioma, we performed gain-of-function assays. The results demonstrated that overexpression of MLK1 or MLK2 significantly inhibited cell proliferation, migration, and invasion in glioma cells (Figures 9c-9i). These findings indicate that MLK1 and MLK2 exert anticancer effects in glioma, which contrasts with the previously reported antitumor effects of ZAK.

Collectively, our study provides valuable insights into the role of MLK1 and MLK2 in glioma progression and highlights their potential as therapeutic targets.

## 4. Discussion

In this study, we have investigated the expression patterns, prognostic values, and molecular functions of MLK1, MLK2, and ZAK in glioma. Our findings indicate that the expression of MLK1 and MLK2 differs significantly between LGG and HGG, and this differential expression correlates with the prognosis of glioma patients. In contrast, the role of ZAK in glioma remains controversial, with previous studies reporting both antitumor and tumorigenic effects. Based on the expression data from the TCGA database, we constructed a new model that showed improved performance in predicting the prognosis of glioma patients. This model, which incorporates the expression levels of MLK1, MLK2, and other relevant genes, provides a valuable tool for clinicians to assess the risk of disease progression and recurrence in glioma patients. Furthermore, we analyzed the association between MLKs and TIICs in glioma. The immune microenvironment plays a crucial role in tumor progression and response to therapy. Our results suggest that MLKs may interact with TIICs to influence the tumor microenvironment and thereby affect glioma progression. Further studies are needed to elucidate the specific mechanisms underlying this interaction.

To gain insights into the molecular functions of MLK1 and MLK2 in glioma, we performed gene ontology (GO) and KEGG analyses. These analyses revealed that MLKs and their coexpressed genes are primarily enriched in the MAPK signaling pathway and protein serine/threonine kinase activity. The MAPK signaling pathway is known to play a critical role in cell proliferation, migration, and invasion, which are hallmarks of cancer. Our in vitro studies demonstrated that overexpression of MLK1 or MLK2 inhibited cell proliferation, migration, and invasion in glioma cells. These findings suggest that MLK1 and MLK2 may exert their anticancer effects in glioma by modulating the MAPK signaling pathway.

MLKs, members of the MAP3K gene family, play a crucial role in activating the MAPK signaling pathway, which is critical for cellular responses to incoming signals. In glioma, recent studies have highlighted the significance of MLKs in various aspects of tumor progression. For instance, Misek et al. [22] and colleagues demonstrated that MLK3 is essential for cell migration and invasion in glioblastoma by activating JNK signaling. Markowitz et al. [30] reported that ZAK protects tumor cells from radiation-induced cell death by regulating cell-cycle arrest after ionizing radiation, suggesting its role in radioresistance. Sung-Hak Kim found that MLK4 is overexpressed in mesenchymal (MES) glioma stem cells and its knockdown suppresses tumorigenesis, self-renewal, motility, and radioresistance. They further showed that MLK4 activates NF-*κ*B signaling in glioma stem cells [32]. Our study revealed that MLKs and their coexpressed genes are mainly enriched in the MAPK signaling pathway and protein serine/threonine kinase activity. This finding suggests that MLKs may play an important role in glioma by regulating the MAPK signaling pathway.

However, further experimental validation is needed to confirm the specific mechanisms of MLKs in glioma. Studies are required to determine which MAPK pathways are primarily activated by MLKs in glioma and how this activation contributes to tumor progression. Additionally, investigating the interaction between MLKs and other components of the MAPK signaling pathway, such as MAP2Ks and MAPKs, will provide further insights into the role of MLKs in glioma.

Tumor immunotherapies have garnered significant attention due to their therapeutic benefits observed across various cancer types [33, 34]. However, in the context of gliomas, several clinical trials investigating immunotherapy have failed to demonstrate improvements in patient survival [35]. TIICs have the potential to either promote or inhibit tumor growth and evasion [36]. The immune system of gliomas comprises various cell types, including macrophages, T cells, NK cells, monocytes, activated DCs, activated mast cells, and eosinophils. Previous studies have suggested that the type and abundance of TIICs within glioma tissues may correlate with patient prognosis [37]. In the present study, we examined the correlation between the expression of MLKs and TIICs. Our results indicate a negative correlation between MLK1 expression and CD4 + T cells in LGG. Similarly, MLK2 expression was negatively correlated with CD8 + T cells in GBM. Furthermore, ZAK expression displayed a positive correlation with dendritic cells in GBM. These findings suggest a potential involvement of MLKs in the immune processes associated with gliomas. However, further research is necessary to substantiate this hypothesis and elucidate the underlying mechanisms.

## 5. Conclusion

In this comprehensive study, we have undertaken a detailed examination of the expression patterns and prognostic significance of MLKs in glioma. Our investigation further explored the potential mechanisms underlying the roles of MLKs in glioma progression, as well as their correlation with TIICs. Notably, we provided evidence of the anticancer effects exerted by MLK1 and MLK2 in glioma. Our findings provide novel insights into the role of MLKs in glioma biology and may pave the way for the development of targeted therapeutic strategies.

## Figures and Tables

**Table 1:** Datasets of *MLKs* in glioma (Oncomine).

Gene	Tumor (cases)	Normal (cases)	Fold change	*t*-Test	*p* value	Dataset
*MLK1*	Glioblastoma (81)	Brain (23)	−5.030	−11.538	1.55E−16	Sun et al.
	Anaplastic astrocytoma (19)	Brain (23)	−3.780	−7.452	4.72E−9	Sun et al.
	Oligodendroglioma (50)	Brain (23)	−3.397	−8.116	1.75E−11	Sun et al.
	Glioblastoma (542)	Brain (10)	−9.198	−18.643	4.61E−9	TCGA
*MLK2*	Anaplastic astrocytoma (19)	Brain (23)	−2.083	−6.405	6.92E−8	Sun et al.
	Glioblastoma (542)	Brain (10)	−2.365	−16.524	8.71E−9	TCGA
*MLK3*	—	—	—	—	—	—
*MLK4*	Anaplastic astrocytoma (19)	Brain (23)	−2.424	−8.336	1.41E−10	Sun et al.
	Oligodendroglioma (50)	Brain (23)	2.656	−8.184	6.91E−12	Sun et al.
	Glioblastoma (81)	Brain (23)	−2.700	−9.896	1.22E−13	Sun et al.
*DLK*	—	—	—	—	—	—
*LZK*	—	—	—	—	—	—
*ZAK*	Glioblastoma (80)	Brain (4)	2.452	13.418	4.18E−7	Murat et al.

Abbreviations: DLK, dual leucine zipper kinase; LZK, leucine zipper kinase; MLKs, mixed lineage kinases; TCGA, The Cancer Genome Atlas; ZAK, sterile alpha motif and leucine zipper containing kinase.

**Table 2:** Subgroup analysis of the prognostic value of *MLKs* in TCGA.

Gene	Variable	LGG	GBM	IDH-wild	IDH-mut
*MLK1*	HR	1.326	0.7053	1.590	1.407
	95% CI	0.9271–1.896	0.4863–1.023	1.168–2.163	0.8815–2.246
	*p* value	0.1223	0.0658	0.0032	0.1523

*MLK2*	HR	1.142	0.8379	1.656	1.071
	95% CI	0.7970–1.635	0.5792–1.212	1.218–2.252	0.6762–1.698
	*p* value	0.4701	0.3478	0.0013	0.7689

*MLK3*	HR	0.8928	0.8808	0.9110	0.9464
	95% CI	0.6178–1.290	0.6091–1.274	0.6687–1.241	0.5863–1.528
	*p* value	0.5458	0.4998	0.5544	0.8217

*DLK*	HR	1.129	0.8797	0.9871	0.8649
	95% CI	0.7907–1.612	0.6085–1.272	0.7256–1.343	0.5471–1.367
	*p* value	0.5046	0.4954	0.9343	0.5345

*LZK*	HR	0.9869	1.049	1.184	0.8669
	95% CI	0.6843–1.423	0.7246–1.519	0.8699–1.612	0.5397–1.393
	*p* value	0.9438	0.7990	0.2826	0.5547

*ZAK*	HR	0.2204	1.090	0.6306	0.6116
	95% CI	0.1516–0.3205	0.7529–1.579	0.4639–0.8572	0.3866–0.9676
	*p* value	< 0.0001	0.6468	0.0032	0.0357

The new signature	HR	0.2071	1.157	0.5778	0.6399
	95% CI	0.1409–0.3045	0.8026–1.669	0.4249–0.7858	0.4044–1.013
	*p* value	< 0.0001	0.4341	0.0005	0.0567

Abbreviations: CI, confidence interval; DLK, dual leucine zipper kinase; HR, hazard ratio; LZK, leucine zipper kinase; MLKs, mixed lineage kinases; TCGA, The Cancer Genome Atlas; ZAK, sterile alpha motif and leucine zipper containing kinase.

**Table 3:** Subgroup analysis of the prognostic value of *MLKs* in CGGA.

Gene	Variable	LGG	GBM	IDH-wild	IDH-mut
*MLK1*	HR	1.882	1.195	1.908	1.880
	95% CI	1.146–3.092	0.7506–1.904	1.226–2.970	1.062–3.328
	*p* value	0.0125	0.4522	0.0042	0.0302

*MLK2*	HR	1.658	1.600	1.785	1.870
	95% CI	1.011–2.720	0.9917–2.582	1.163–2.740	1.065–3.284
	*p* value	0.0453	0.0541	0.0081	0.0293

*MLK3*	HR	0.7684	0.8764	0.6799	0.7847
	95% CI	0.4670–1.264	0.5399–1.423	0.4356–1.061	0.4450–1.383
	*p* value	0.2998	0.5935	0.0896	0.4019

*MLK4*	HR	1.156	0.8620	1.652	1.221
	95% CI	0.6917–1.933	0.5299–1.402	1.073–2.545	0.6719–2.219
	*p* value	0.5796	0.5499	0.0227	0.5123

*DLK*	HR	0.8858	1.015	1.063	1.081
	95% CI	0.5406–1.451	0.6176–1.667	0.6878–1.642	0.6150–1.900
	*p* value	0.6302	0.9541	0.7837	0.7865

*LZK*	HR	0.9643	0.8654	0.5389	0.9559
	95% CI	0.5851–1.589	0.5428–1.380	0.3486–0.8330	0.5350–1.708
	*p* value	0.8866	0.5437	0.0054	0.8790

*ZAK*	HR	0.2583	0.7716	0.7602	0.7889
	95% CI	0.1414–0.4720	0.4838–1.231	0.4987–1.159	0.4389–1.418
	*p* value	< 0.0001	0.2762	0.2024	0.4280

The new signature	HR	0.2626	0.6641	0.7148	0.8321
	95% CI	0.1427–0.4832	0.4129–1.068	0.4690–1.089	0.8321
	*p* value	< 0.0001	0.0912	0.1183	0.5417

Abbreviations: CGGA, Chinese Glioma Genome Atlas; CI, confidence interval; DLK, dual leucine zipper kinase; HR, hazard ratio; LZK, leucine zipper kinase; MLKs, mixed lineage kinases; ZAK, sterile alpha motif and leucine zipper containing kinase.

**Table 4:** GO terms enrichment (including top five MF, top five BP, and top two CC).

Category	Term2	Count	*p* value	Genes	Fold enrichment	FDR
GOTERM_MF_DIRECT	GO:0004674~protein serine/threonine kinase activity	14	2.49E−16	*ACVRL1*, *CSNK2A1*, *TNK2*, *NEK7*, *KSR2*, *TGFBR1*, *DSTYK*, *ACVR1C*, *IRAK1*, *MAP3K10*,*MAP3K9*, *MAP3K13*, *MAP3K11*, *MAP3K12*	26.18949	1.24E−14

GOTERM_MF_DIRECT	GO:0005524~ATP binding	19	2.29E−15	*BLK*, *ACVRL1*, *KSR1*, *CSNK2A1*, *TNK2*, *NEK7*, *TNK1*, *KSR2*, *TGFBR1*, *TGFBR2*, *DSTYK*, *ACVR1C*, *IRAK1*, *MAP3K10*, *ABL2*, *MAP3K9*, *MAP3K13*, *MAP3K11*, *MAP3K12*	8.93921	5.72E−14

GOTERM_MF_DIRECT	GO:0004672~protein kinase activity	12	3.72E−13	*CSNK2A1*, *IRAK1*, *KSR1*, *TNK2*, *NEK7*, *MAP3K10*, *ABL2*, *MAP3K9*, *KSR2*, *TGFBR1*, *MAP3K11*, *MAP3K12*	23.51114	6.20E−12

GOTERM_MF_DIRECT	GO:0004713~protein tyrosine kinase activity	8	3.55E−10	*BLK*, *DSTYK*, *TNK2*, *TNK1*, *MAP3K10*, *ABL2*, *MAP3K9*, *MAP3K11*	42.30827	4.43E−09

GOTERM_MF_DIRECT	GO:0004709~MAP kinase kinase kinase activity	4	3.35E−06	*MAP3K10*, *MAP3K9*, *MAP3K13*, *MAP3K12*	127.88636	3.35E−05

GOTERM_BP_DIRECT	GO:0006468~protein phosphorylation	13	1.44E−13	*ACVRL1*, *CSNK2A1*, *NEK7*, *TNK1*, *KSR2*, *TGFBR1*, *TGFBR2*, *ACVR1C*, *IRAK1*, *MAP3K9*, *MAP3K13*, *MAP3K11*, *MAP3K12*	19.94664	4.23E−11

GOTERM_BP_DIRECT	GO:0007254~JNK cascade	6	5.55E−09	*IRAK1*, *MAP3K10*, *MAPK8IP2*, *MAP3K13*, *MAP3K11*, *MAP3K12*	85.67347	8.16E−07

GOTERM_BP_DIRECT	GO:0046777~protein autophosphorylation	7	9.25E−08	*IRAK1*, *TNK1*, *MAP3K10*, *MAP3K9*, *MAP3K13*, *MAP3K11*, *MAP3K12*	28.47481	9.06E−06

GOTERM_BP_DIRECT	GO:0018107~peptidyl-threonine phosphorylation	4	1.84E−05	*MAP3K10*, *TGFBR1*, *TGFBR2*, *MAP3K12*	73.64912	0.001204

GOTERM_BP_DIRECT	GO:0038083~peptidyl-tyrosine autophosphorylation	4	2.15E−05	*BLK*, *TNK2*, *TNK1*, *ABL2*	69.96667	0.001204

GOTERM_CC_DIRECT	GO:0005622~intracellular	10	1.85E−05	*KSR1*, *MAP3K10*, *MAP3K9*, *ZNF567*, *KSR2*, *MAPK8IP2*, *MAP3K13*, *TGFBR1*, *MAP3K11*, *MAP3K12*	5.70070	0.001295

GOTERM_CC_DIRECT	GO:0031234~extrinsic component of cytoplasmic side of plasma membrane	4	8.34E−05	*BLK*, *TNK2*, *TNK1*, *ABL2*	44.66667	0.00292

Abbreviations: BP, biological processes; CC, cellular component; MF, molecular functions.

**Table 5:** Top five of KEGG pathway enrichment.

Category	Term2	Count	*p* Value	Genes	Fold enrichment	FDR
KEGG_PATHWAY	hsa04010:MAPK signaling pathway	6	2.49E−05	*MAPK8IP2*, *MAP3K13*, *TGFBR1*, *MAP3K11*, *TGFBR2*, *MAP3K12*	13.59486	7.73E−04
KEGG_PATHWAY	hsa04520:adherens junction	3	0.00544	*CSNK2A1*, *TGFBR1*, *TGFBR2*	24.22183	0.077963
KEGG_PATHWAY	hsa04350:TGF-β signaling pathway	3	0.007545	*ACVR1C*, *TGFBR1*, *TGFBR2*	20.47321	0.077963
KEGG_PATHWAY	hsa05142:Chagas disease (American trypanosomiasis)	3	0.011392	*IRAK1*, *TGFBR1*, *TGFBR2*	16.53606	0.088285
KEGG_PATHWAY	hsa04014:Ras signaling pathway	3	0.048617	*KSR1*, *ABL2*, *KSR2*	7.60951	0.301427

Abbreviations: KEGG, Kyoto Encyclopedia of Genes and Genomes; MAPK, mitogen-activated protein kinase; TGF-β, transforming growth factor beta.

## Data Availability

The datasets used and/or analyzed during the current study are available from the corresponding author upon reasonable request.

## Supporting Information

Supporting Information Table S1: primers for RT-qPCR. Table S2: Spearman's correlations of mRNA expression of each member in MLKs in TCGA and CGGA. Table S3: the association between MLKs and clinicopathological characteristics in TCGA. Table S4: the association between MLKs and clinicopathological characteristics in CGGA.
