# Supplementary material for: The Expression Profiles and Clinical Significance of Mixed Lineage Kinases in Glioma
Source: Mediators Inflamm. 2024 Nov 21;2024:5521016. doi: 10.1155/2024/5521016 (PMC11604285; doi:10.1155/2024/5521016)
Supplement: Supporting Information — Table S1: primers for RT-qPCR. Table S2: Spearman's correlations of mRNA expression of each member in MLKs in TCGA and CGGA. Table S3: the association between MLKs and clinicopathological characteristics in TCGA. Table S4: the association between MLKs and clinicopathological characteristics in CGGA. [file 5521016.f1.docx]

**Supplemental material Table 1. Primers for RT-qPCR**

| genes | Forward primer sequence(5’-3’) | Reverse primer sequence(5’-3’) |
| --- | --- | --- |
| MLK1 | gCgATgAAATTgTCgTgTATgA | gTgggAgTCAgAgATTggTTAg |
| MLK2 | CCCAAAgAAggAAgAACTggTC | CTTgATgACCACTgTTTgCTTC |

**Supplemental material Table 2. Spearman’s correlations of mRNA expression of each member**

**in MLKs in TCGA and CGGA**

| **CGGA** | | | | **TCGA** | | | |
| --- | --- | --- | --- | --- | --- | --- | --- |
| gene1 | gene2 | cor_spearman | p_spearman | gene1 | gene2 | cor_spearman | p_spearman |
| DLK | LZK | -0.016042892 | 0.0440199 | DLK | LZK | 0.31713119 | 0 |
| DLK | ZAK | 0.068746035 | 7.988E-06 | DLK | ZAK | -0.1539513 | 2.2204E-15 |
| LZK | ZAK | -0.1128506 | 0.0440199 | LZK | ZAK | -0.0315605 | 0.17305216 |
| MLK1 | MLK2 | 0.528187875 | 0 | MLK1 | MLK2 | 0.47890683 | 0 |
| MLK1 | MLK3 | -0.299447638 | 4.5987E-06 | MLK1 | MLK3 | -0.2555996 | 3.4872E-11 |
| MLK1 | MLK4 | 0.599906421 | 0.49640366 | MLK1 | DLK | 0.21405207 | 0 |
| MLK1 | DLK | 0.045472255 | 0 | MLK1 | LZK | 0.61937351 | 0.10340414 |
| MLK1 | LZK | -0.051599309 | 0.81792266 | MLK1 | ZAK | -0.1277136 | 3.4872E-11 |
| MLK1 | ZAK | -0.233954747 | 0.98571203 | MLK2 | MLK3 | 0.06383714 | 3.404E-08 |
| MLK2 | MLK3 | -0.181358323 | 0 | MLK2 | DLK | 0.29339885 | 0.0010823 |
| MLK2 | MLK4 | 0.292154678 | 0.44016138 | MLK2 | LZK | 0.28327776 | 2.065E-14 |
| MLK2 | DLK | -0.001539394 | 0.00625693 | MLK2 | ZAK | -0.3227867 | 0.10340414 |
| MLK2 | LZK | -0.015398326 | 2.7705E-11 | MLK3 | DLK | 0.06755163 | 0 |
| MLK2 | ZAK | -0.42416521 | 0.19847481 | MLK3 | LZK | -0.3036822 | 1.6942E-13 |
| MLK3 | MLK4 | -0.134100647 | 0.0003897 | MLK3 | ZAK | -0.0534225 | 0.0847905 |
| MLK3 | DLK | 0.001197882 | 7.988E-06 |  |  |  |  |
| MLK3 | LZK | -0.085855449 | 4.5987E-06 |  |  |  |  |
| MLK3 | ZAK | 0.159579251 | 0.01634557 |  |  |  |  |
| MLK4 | DLK | 0.173381487 | 0.98163923 |  |  |  |  |
| MLK4 | LZK | -0.150834635 | 0.00625693 |  |  |  |  |
| MLK4 | ZAK | 0.090973794 | 0 |  |  |  |  |

**Supplemental material Table 3. The association between MLKs and clinicopathological characteristics in TCGA**

| Characteristics | n | MLK1 | | P Value^a^ | MLK2 | | P Value^a^ | MLK3 | | P Value^a^ | DLK | | P Value^a^ | LZK | | P Value^a^ | ZAK | | P Value^a^ | The new model | | P Value^a^ |
| --- | --- | --- | --- | --- | --- | --- | --- | --- | --- | --- | --- | --- | --- | --- | --- | --- | --- | --- | --- | --- | --- | --- |
|  |  | Low | High |  | Low | High |  | Low | High |  | Low | High |  | Low | High |  | Low | High |  | Low | High |  |
| Age |  |  |  | 0.0521 |  |  | 0.0336 |  |  | 0.2077 |  |  | 0.0780 |  |  | 0.0108 |  |  | < 0.0001 |  |  | < 0.0001 |
| <60 | 499 | 244 | 255 |  | 263 | 236 |  | 272 | 227 |  | 222 | 277 |  | 217 | 282 |  | 302 | 197 |  | 316 | 183 |  |
| ≥60 | 152 | 88 | 64 |  | 95 | 57 |  | 74 | 78 |  | 80 | 72 |  | 84 | 68 |  | 28 | 124 |  | 32 | 120 |  |
| Gender |  |  |  | 0.1893 |  |  | 0.2370 |  |  | 0.7168 |  |  | 0.9277 |  |  | 0.5252 |  |  | 0.6838 |  |  | 0.2122 |
| Female | 279 | 134 | 145 |  | 146 | 133 |  | 146 | 133 |  | 130 | 149 |  | 125 | 154 |  | 144 | 135 |  | 157 | 122 |  |
| male | 372 | 198 | 174 |  | 212 | 160 |  | 200 | 172 |  | 172 | 200 |  | 176 | 196 |  | 186 | 186 |  | 191 | 181 |  |
| WHO grades |  |  |  | < 0.0001 |  |  | < 0.0001 |  |  | 0.0956 |  |  | 0.0265 |  |  | < 0.0001 |  |  | < 0.0001 |  |  | < 0.0001 |
| LGG | 504 | 244 | 260 |  | 239 | 265 |  | 259 | 245 |  | 222 | 282 |  | 211 | 293 |  | 320 | 184 |  | 338 | 166 |  |
| GBM | 147 | 108 | 39 |  | 119 | 28 |  | 87 | 60 |  | 80 | 67 |  | 90 | 57 |  | 10 | 137 |  | 10 | 137 |  |
| IDH type |  |  |  | 0.0081 |  |  | < 0.0001 |  |  | 0.5194 |  |  | 0.0020 |  |  | 0.0016 |  |  | < 0.0001 |  |  | < 0.0001 |
| Wild type | 254 | 146 | 108 |  | 165 | 89 |  | 139 | 115 |  | 137 | 117 |  | 137 | 117 |  | 34 | 220 |  | 38 | 216 |  |
| Mut type | 397 | 186 | 211 |  | 193 | 204 |  | 207 | 190 |  | 165 | 232 |  | 164 | 233 |  | 296 | 101 |  | 311 | 86 |  |

**Supplemental material Table 4. The association between MLKs and clinicopathological characteristics in CGGA**

| Characteristics | n | MLK1 | | P Value^a^ | MLK2 | | P Value^a^ | MLK3 | | P Value^a^ | MLK4 | | P Value^a^ | DLK | | P Value^a^ | LZK | | P Value^a^ | ZAK | | P Value^a^ | The new signature | | P Value^a^ |  |
| --- | --- | --- | --- | --- | --- | --- | --- | --- | --- | --- | --- | --- | --- | --- | --- | --- | --- | --- | --- | --- | --- | --- | --- | --- | --- | --- |
|  |  | Low | High |  | Low | High |  | Low | High |  | Low | High |  | Low | High |  | Low | High |  | Low | High |  | Low | High |  | |
| Age |  |  |  | 0.0012 |  |  | 0.0039 |  |  | 0.0147 |  |  | 0.3170 |  |  | 0.2795 |  |  | 0.4803 |  |  | 0.0006 |  |  | 0.0353 | |
| <60 | 194 | 129 | 65 |  | 111 | 83 |  | 123 | 71 |  | 127 | 67 |  | 118 | 76 |  | 104 | 90 |  | 122 | 72 |  | 117 | 77 |  | |
| ≥60 | 28 | 27 | 1 |  | 24 | 4 |  | 11 | 17 |  | 21 | 7 |  | 20 | 8 |  | 17 | 11 |  | 8 | 20 |  | 11 | 17 |  | |
| Gender |  |  |  | 0.5503 |  |  | 0.8424 |  |  | 0.9330 |  |  | 0.7691 |  |  | 0.1722 |  |  | 0.1080 |  |  | 0.1765 |  |  | 0.2008 | |
| Female | 84 | 61 | 23 |  | 50 | 34 |  | 51 | 33 |  | 57 | 27 |  | 57 | 27 |  | 40 | 44 |  | 54 | 30 |  | 53 | 31 |  | |
| male | 138 | 95 | 43 |  | 84 | 54 |  | 83 | 55 |  | 91 | 47 |  | 81 | 57 |  | 81 | 57 |  | 76 | 62 |  | 75 | 63 |  | |
| WHO grades |  |  |  | 0.0007 |  |  | 0.0010 |  |  | 0.0086 |  |  | 0.6254 |  |  | 0.0119 |  |  | 0.5254 |  |  | < 0.0001 |  |  | < 0.0001 | |
| LGG | 137 | 85 | 52 |  | 71 | 66 |  | 92 | 45 |  | 93 | 44 |  | 94 | 43 |  | 73 | 64 |  | 102 | 35 |  | 98 | 39 |  | |
| GBM | 85 | 71 | 14 |  | 63 | 22 |  | 42 | 43 |  | 55 | 30 |  | 44 | 41 |  | 49 | 36 |  | 28 | 57 |  | 30 | 55 |  | |
| IDH type |  |  |  | 0.0599 |  |  | 0.0246 |  |  | 0.0072 |  |  | 0.1066 |  |  | 0.0069 |  |  | 0.0407 |  |  | < 0.0001 |  |  | < 0.0001 | |
| Wild type | 109 | 83 | 26 |  | 75 | 34 |  | 56 | 53 |  | 67 | 42 |  | 58 | 51 |  | 67 | 42 |  | 29 | 80 |  | 30 | 79 |  | |
| Mut type | 113 | 73 | 40 |  | 60 | 51 |  | 78 | 35 |  | 81 | 32 |  | 80 | 33 |  | 54 | 59 |  | 101 | 12 |  | 98 | 15 |  | |

^a^*p* value for χ^2^ test
